# Deep Dermal Dilemma: A Case Report on Majocchi’s Granuloma After Topical Steroid Use

**DOI:** 10.7759/cureus.71807

**Published:** 2024-10-18

**Authors:** Renuka Devi Veerakumar, Aravind Baskar Murthy, Priya Cinna T Durai, Murali Narasimhan

**Affiliations:** 1 Dermatology, Venereology, and Leprosy, Sri Ramaswamy Memorial (SRM) Medical College Hospital and Research Centre, Chennai, IND

**Keywords:** deep dermatophyte infection, fungal infection, majocchi’s granuloma, nodular granulomatous perifolliculitis, terbinafine

## Abstract

Majocchi’s granuloma represents a unique dermatological entity characterized by the invasion of dermatophytes into the dermis and subcutaneous tissue, typically following trauma or topical corticosteroids, most commonly caused by *Trichophyton rubrum*. The unrestrained use of over-the-counter topical steroids has led to the rise of Majocchi’s granuloma in the past few years. A 57-year-old male presented with complaints of itchy skin lesions over his right ankle for 20 days. There was a history of over-the-counter topical steroid application, following which the lesions exacerbated. Clinical examination showed multiple ill-defined erythematous nodules, 2-3 cm in size, with serosanguinous discharge associated with right ankle swelling. A single annular hyperpigmented scaly patch was also noted over the right leg below the knee. Potassium hydroxide (KOH) examination was positive for fungal hyphae. Histopathological examination (HPE) of the nodule showed features suggestive of Majocchi's granuloma. Fungal culture was performed, which showed growth of *Trichophyton rubrum*. The patient was started on oral terbinafine 250 mg once daily and topical luliconazole cream twice daily topical application, which resolved the lesions in three weeks, but the patient was advised to continue the treatment for a total duration of two months.

## Introduction

Majocchi’s granuloma is a rare dermatological condition characterized by the invasion of dermatophytes into the dermis and subcutaneous tissue, typically following trauma or topical corticosteroids [1]. It is most commonly caused by *Trichophyton rubrum *and can involve any hair-bearing area such as the scalp, face, or extremities [2]. Majocchi's granuloma represents a paradox, as dermatophytes usually cause superficial infections limited to stratum corneum. The deeper involvement of dermis makes the disease clinically and pathologically unique among dermatophyte infections [1,2]. The clinical features differ between immunocompetent and immunocompromised individuals [3]. The unrestrained use of over-the-counter topical steroids has led to the rise of Majocchi’s granuloma in the past few years. In this report, we present a rare case of topical steroid-induced Majocchi's granuloma, emphasizing the importance of considering deep dermatophyte infections in patients presenting with atypical nodules over the legs.

## Case presentation

**Figure 1:**
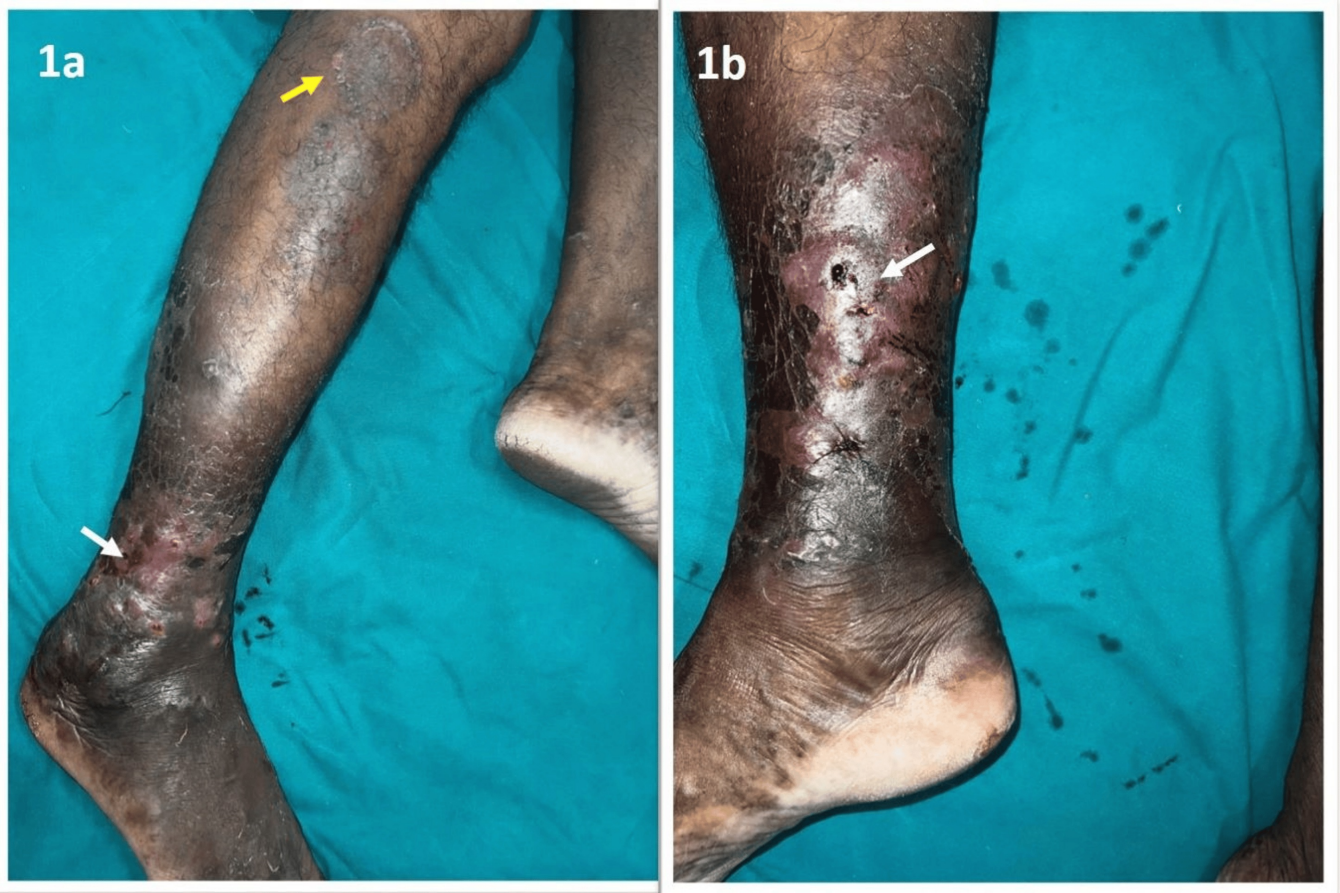
(a, b) Multiple ill-defined erythematous nodules, 2-3 cm in size, indicated by white arrows, with serosanguinous discharge associated with right ankle swelling and a single annular hyperpigmented scaly patch, 4 x 4 cm, indicated by the yellow arrow, below the knee.

A 57-year-old male presented with complaints of itchy erythematous skin lesions over his right ankle for 20 days. There was a history of over-the-counter topical steroid application from the next day onwards till the day of presentation. Though initially, the erythema seemed to be reduced, after five days of topical steroid application, the lesions aggravated with the development of nodules and discharge. Clinical examination showed multiple ill-defined erythematous nodules, 2-3 cm in size, with serosanguinous discharge associated with right ankle swelling. A single annular hyperpigmented scaly patch was also noted over the right leg below the knee (Figure 1).

**Figure 2:**
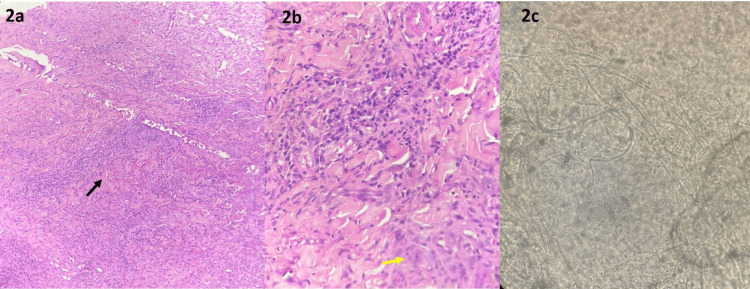
Histopathological examination of the nodule showing Langhans giant cells, indicated by the yellow arrow, and mixed inflammatory infiltrate of lymphocytes, macrophages, epithelioid cells, neutrophils, and plasma cells with focal necrosis in deep dermis, indicated by the black arrow, suggestive of Majocchi's granuloma (H&E stain) in (a) x10 magnification and (b) x40 magnification (c). Potassium hydroxide examination of the discharge showing fungal hyphae (x10).

The patient was afebrile and did not have associated regional lymphadenopathy. The systemic examination was also found to be normal. Potassium hydroxide (KOH) examination was positive for fungal hyphae. Histopathological examination (HPE) of the nodule showed mixed inflammatory infiltrate of lymphocytes, macrophages, epithelioid cells, neutrophils, and plasma cells in the deep dermis, with few Langhans giant cells suggestive of Majocchi's granuloma (Figure 2).

**Figure 3:**
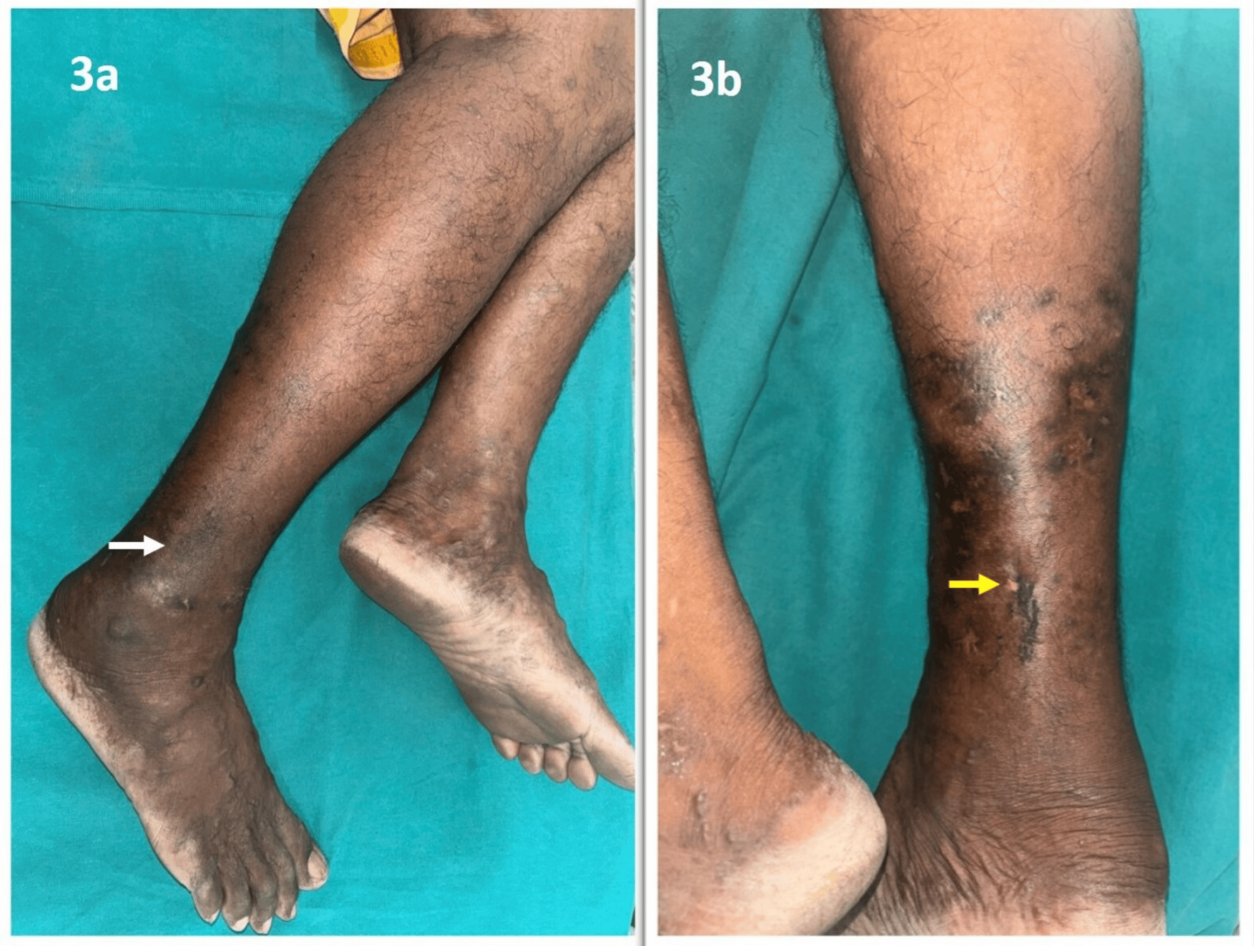
(a, b) Ill-defined hyperpigmented patches and atrophic scars, indicated by white and yellow arrows, respectively, following resolution of lesions in three weeks over the right leg.

The discharge was sent for fungal culture sensitivity, which showed growth of *Trichophyton rubrum*. Based on the clinical, microscopy, and histopathological features, the patient was diagnosed with Majocchi’s granuloma. The patient was started on oral terbinafine 250 mg once daily and topical luliconazole cream twice daily topical application, which resolved the lesions in three weeks, but the patient was advised to continue the treatment for a total duration of two months (Figure 3).

## Discussion

Majocchi’s granuloma was first described by Domenico Majocchi in 1883 in Italy, caused by *Trichophyton tonsurans *[4,5]. Majocchi’s granuloma is also known as Majocchi’s trichophytic granuloma, dermatophytic granuloma, nodular granulomatous perifolliculitis, and granuloma tricofitico [4,5]. It is a deep dermatophytic infection most commonly caused by *Trichophyton rubrum*. The other causes are described in Table 1 [4,6-10].

**Table 1:** Causative organisms for Majocchi’s granuloma [4,6-10]

Organisms causing Majocchi’s granuloma
*Trichophyton rubrum* (most common)
*Trichophyton mentagrophytes *(isolated from rodents)
*Trichophyton violaceum* (isolated from CARD9 deficiency patients)
*Trichophyton tonsurans* (usually seen in AIDS patients)
*Trichophyton verrucosum *(isolated from cattle)
*Microsporum canis *(isolated from cats)
Trichophyton interdigitale
Microsporum audouinii
Microsporum ferrugineum
Non-dermatophytes (*Aspergillus*, *Alternaria*, and *Candida albicans*)

The predisposing factors include chronic untreated dermatophytosis, impaired skin barrier secondary to trauma, animal exposure, immunocompromised patients, long-term use of steroids, chemotherapeutic drugs, use of adalimumab in organ transplantation patients, and BRAF inhibitors (vemurafenib) [6]. A study by Boral et al. showed that topical steroid application for erythematous squamous lesions is the most common predisposing factor for the development of Majocchi’s granuloma accounting for 55% [6]. CARD 9 deficiency is the known risk factor for Majocchi’s granuloma, especially in the Mediterranean region, with the most common organism being *Trichophyton violaceum* [11].

The most important factor triggering the pathogenesis of the disease is physical trauma such as scratching or shaving, leading to fungal invasion. Fungal lysin motif (LysM) domain-associated proteins mask the fungal cell wall chitin, thus protecting it from host immunity. The genes encoding the key components of the glyoxylate pathway and secretion of sulfite destroy components of the skin [6]. The mean age of patients is 38 years, and the mean disease duration of nine months (range: 3 to 60 months) [6]. Females are more affected than males, with a ratio of 3:1 [4]. The disease presents differently in immunocompetent and immunocompromised individuals, as discussed in Table 2 [4,6,12].

**Table 2:** Clinical features of Majocchi’s granuloma in immunocompetent and immunocompromised individuals [4,6,12]

Features	Immunocompetent	Immunocompromised
Morphology	Papulopustular perifollicular lesions, deep papules, and pustules	Indurated plaques with erythematous subcutaneous nodules
Predominant form	Plaques	Nodules
Predisposing factors	Penetrating trauma	Patients on immunosuppressants
Common site	Lower extremities	Upper extremities
Associated conditions	Atopic dermatitis, topical steroid use, shaving/scratching	Leukemia/lymphoma, autoimmune diseases, chemotherapy, post-organ transplant, CARD9 deficiency

Majocchi’s granuloma can present as papules, patches, pustules, verrucous plaque, or nodules [7,13]. The most common involved sites are face and lower extremities in immunocompetent and immunocompromised individuals, respectively, with the frequency of facial involvement increasing in the recent past [6]. Palmoplantar hyperkeratosis and erythroderma have been associated with Majocchi’s granuloma in an AIDS patient, caused by *Trichophyton tonsurans* [14]. Post-inflammatory pigmentation, alopecia, and scarring occur as a sequela of Majocchi’s granuloma [6]. The disease progresses through three clinical phases, based on morphology namely plaques, nodules, and degeneration [4]. The three main criteria to diagnose Majocchi’s granuloma are lesions caused by dermatophytes, perifollicular granulomatous inflammation in histopathological examination, and the presence of dermatophytes in superficial layers and dermis [14,15]. Majocchi's granuloma is usually misdiagnosed initially due to the presence of numerous diseases, with erythematous nodules affecting the extremities especially erythema nodosum, mycetoma, and furunculosis. The differential diagnosis for Majocchi’s granuloma is explained in Table 3 [4,6].

**Table 3:** Differential diagnosis of Majocchi’s granuloma [4,6]

Differential diagnosis
Mycobacterial infections
Deep fungal infections
Folliculitis
Erythema nodosum
Disseminated toxoplasmosis
Cutaneous leishmaniasis
Face and scalp: granulomatous rosacea, granuloma faciale, tinea capitis, kerion celsi
Malignancies: Kaposi sarcoma, lymphoma

The KOH examination shows fungal hyphae and spores, but this cannot differentiate between superficial and deep dermatophytosis [16]. Therefore, histopathological examination is considered the gold standard, which shows perifollicular granulomatous inflammation (granulomatous infiltrate of lymphoid cells, macrophages, epithelioid cells, multinucleated giant cells, neutrophils) [6]. Special stains such as Gomori methenamine silver (GMS) and periodic acid Schiff (PAS) stains aid in the confirmation of diagnosis, with GMS being superior to PAS [6]. Examination of histopathological specimens under an immunofluorescence microscope reveals fungi as auto-fluorescent particles [6]. A negative result in microscopy cannot exclude the diagnosis of Majocchi’s granuloma [12]. Fungal culture can be performed for further confirmation of causative fungi [6]. Molecular methods such as internal transcribed spacer sequencing can also be performed for the identification of fungus, especially in immunocompromised individuals [6]. Combination of culture and microscopy has a higher chance of detecting fungal organisms [6].

The treatment options include oral terbinafine (250 mg/day), itraconazole (100-200 mg/day), griseofulvin (250-500 mg/day), voriconazole, posaconazole, potassium iodide, local X-radiation, and topical 2-dimethylamino-6-(beta-diethylaminoethyl)-benzothiazole, with the most preferred drug being terbinafine [6]. The duration of treatment varies from one to six months, and treatment must be continued until the lesions have healed completely [6]. Cryotherapy has been tried in a few refractory cases [12,17].

## Conclusions

The diagnosis of Majocchi’s granuloma is challenging due to the varied differential diagnoses requiring confirmation with the gold standard histopathological examination. Clinicians should consider Majocchi’s granuloma as a diagnosis for patients with nodular or granulomatous skin lesions especially following topical steroid use, immunosuppression, or trauma. The rampant use of topical steroids requires stringent policies to curb the availability of over-the-counter steroids and a call to action for healthcare providers for health education on topical steroid misuse. Early diagnosis and prompt treatment of superficial fungal infections play a pivotal role in preventing deeper infections such as Majocchi's granuloma.
